# In Vitro Fabrication of Hybrid Bone/Cartilage Complex Using Mouse Induced Pluripotent Stem Cells

**DOI:** 10.3390/ijms21020581

**Published:** 2020-01-16

**Authors:** Phoonsuk Limraksasin, Takeru Kondo, Maolin Zhang, Hiroko Okawa, Thanaphum Osathanon, Prasit Pavasant, Hiroshi Egusa

**Affiliations:** 1 Division of Molecular and Regenerative Prosthodontics, Tohoku University Graduate School of Dentistry, Sendai, Miyagi 980-8575, Japan; 2 Weintraub Center for Reconstructive Biotechnology, UCLA (University of California, Los Angeles) School of Dentistry, Los Angeles, CA 90095-1668, USA; 3 Center of Excellence for Regenerative Dentistry and Department of Anatomy, Faculty of Dentistry, Chulalongkorn University, Bangkok 10330, Thailand; 4 Center for Advanced Stem Cell and Regenerative Research, Tohoku University Graduate School of Dentistry, Sendai, Miyagi 980-8575, Japan

**Keywords:** bioengineering, iPS cells, mechanical stimuli, osteochondroral tissue, self-organization

## Abstract

Cell condensation and mechanical stimuli play roles in osteogenesis and chondrogenesis; thus, they are promising for facilitating self-organizing bone/cartilage tissue formation in vitro from induced pluripotent stem cells (iPSCs). Here, single mouse iPSCs were first seeded in micro-space culture plates to form 3-dimensional spheres. At day 12, iPSC spheres were subjected to shaking culture and maintained in osteogenic induction medium for 31 days (Os induction). In another condition, the osteogenic induction medium was replaced by chondrogenic induction medium at day 22 and maintained for a further 21 days (Os-Chon induction). Os induction produced robust mineralization and some cartilage-like tissue, which promoted expression of osteogenic and chondrogenic marker genes. In contrast, Os-Chon induction resulted in partial mineralization and a large area of cartilage tissue, with greatly increased expression of chondrogenic marker genes along with *osterix* and *collagen 1a1*. Os-Chon induction enhanced mesodermal lineage commitment with brachyury expression followed by high expression of lateral plate and paraxial mesoderm marker genes. These results suggest that combined use of micro-space culture and mechanical stimuli facilitates hybrid bone/cartilage tissue formation from iPSCs, and that the bone/cartilage tissue ratio in iPSC constructs could be manipulated through the induction protocol.

## 1. Introduction

The treatment of articular cartilage and osteochondral defects remains challenging, but cell-based tissue engineering is expected to enable the regeneration of osteochondral tissue [1]. Generation of osteochondral tissue is also important for bone tissue engineering. Although bone has innate potential for regeneration after injury, excessive bone defects exceed the capacity for natural bone healing, resulting in impairment of bone formation [2]. Therefore, critical-size bone defects require additional intervention, especially tissue-engineered grafts, to facilitate the healing process. Currently the dominant approaches for enhancing bone formation include recapitulation of intramembranous ossification such as the use of autologous bone grafts [3] and allogeneic bone tissue transplantation [4]. In contrast, most severe bone injuries heal by remodeling of hypertrophic cartilaginous anlage, known as endochondral ossification. Endochondral ossification can promote angiogenesis and bone tissue formation. In addition, chondrocytes can survive with low oxygen tension and poor nutrients [5]. Freeman and McNamara [6] reported that chondrocytes produce extracellular matrix (ECM) to maintain the space and load bearing for bone formation. Therefore, endochondral ossification-based strategies are promising to provide sufficient bone regeneration in large defects.

Induced pluripotent stem cells (iPSCs) derived from the reprogramming of somatic cells have self-renewal properties, i.e., they can proliferate and still maintain their pluripotency. Furthermore, iPSCs can differentiate into all three germ layers: endoderm, mesoderm and ectoderm. The self-organizing potential of iPSCs contributes to three-dimensional (3D) tissue/organ formation without use of scaffolds [7]. Therefore, iPSCs could be a promising cell source for in vitro tissue engineering. In addition, iPSCs have an intrinsic ability to form cell aggregates, so-called embryoid bodies (EB), by floating culture in growth medium. Efficient production of EBs using culture devices such as micro-space culture plates [8] would be advantageous to induce cell condensation, which is an essential step in the self-organizing process for skeletal formation [9].

Mechanical stimuli affect in vivo osteogenesis and chondrogenesis [10]. Several studies have supported the role of mechanical force in enhancing osteogenic [11,12] and chondrogenic differentiation [13,14] of stem cells. Previous studies demonstrated osteogenic differentiation induction of mouse iPSCs [15] to form osseous-like tissue constructs [16] using gentle shaking culture; however, the use of mechanical stimuli in the fabrication of osteochondral complexes has not been reported.

We hypothesized that combined use of micro-space culture and mechanical stimulation could facilitate fabrication of osteochondral constructs by enhancing osteochondrogenic differentiation of iPSCs. The objectives of this study were to establish a platform for self-organizing induction of iPSCs toward osteochondrogenic cells to fabricate osteochondral organoids and to manipulate the bone and cartilage ratio of the construct.

## 2. Results

### 2.1. Effects of Osteogenic (Os) and Osteo-Chondrogenic (Os-Chon) Induction on Osteochondrogenic Differentiation of 3D-iPSC Constructs

#### 2.1.1. Expression of Osteogenic Marker Genes

**Figure 1:**
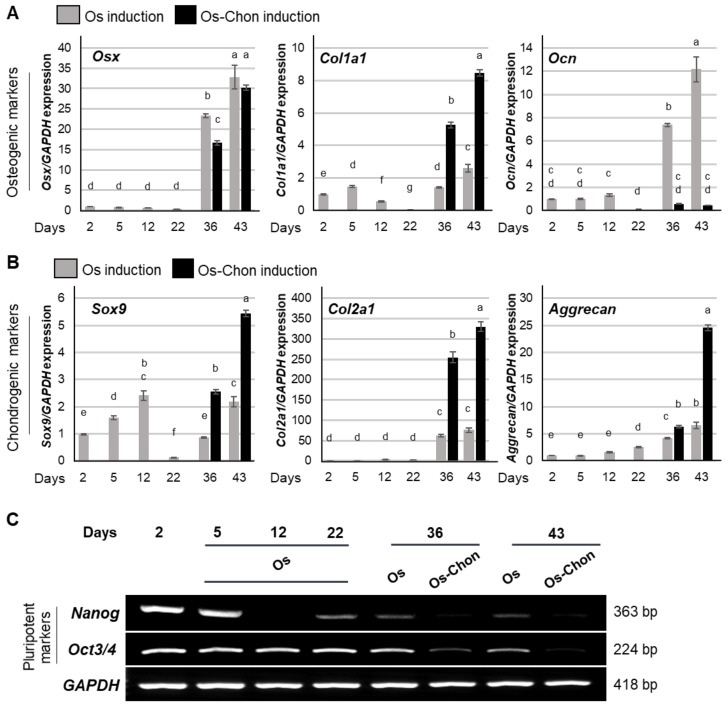
Effects of osteogenic (Os) induction and osteo-chondrogenic (Os-Chon) induction on osteochondrogenic differentiation of three-dimensional-induced pluripotent stem cells (3D-iPSC) constructs. Expression of (**A**) osteogenic marker genes [*osterix* (*Osx*), *collagen 1a1* (*Col1a1*) and *osteocalcin* (*Ocn*)] and (**B**) chondrogenic marker genes [*Sox9*, *collagen 2a1* (*Col2a1*) and *aggrecan*] were evaluated using real-time reverse transcription polymerase chain reaction (RT-PCR) on day 2 (the day when the induction commenced), on days 5, 12, and 22 (Os induction; same induction protocol as Os-Chon induction during this period) and on days 36 and 43 (Os or Os-Chon induction). The data represent the mean ± SD (*n* = 3). Different letters indicate significant differences between groups (*p* < 0.05, one-way analysis of variance (ANOVA) with Tukey’s multiple comparison test). (**C**) Expression of pluripotency marker genes, *Nanog* and *Oct3/4*, was evaluated by semi-quantitative RT-PCR analysis. *GAPDH* expression was used as an internal control.

The expression of *osterix* (*Osx*) was significantly increased after day 36, and was further increased until 43 days, by both Os and Os-Chon induction (Figure 1A). After day 36, expression of *Osx* was higher with Os induction than with Os-Chon induction. Expression of *collagen 1a1* (*Col1a1*) was slightly increased at day 5 by Os induction (Figure 1A). Expression of *Col1a1* decreased during the early to middle Os induction period (day 12–22) and then increased after 36 days of both Os and Os-Chon induction. After day 36, expression of *Col1a1* with Os-Chon induction was significantly higher than that with Os induction. Expression of *osteocalcin* (*Ocn*) decreased at day 22, followed by marked upregulation with Os induction, whereas the expression remained low with Os-Chon induction after 36 days (Figure 1A).

#### 2.1.2. Expression of Chondrogenic Marker Genes

*Sox9* expression gradually increased during the early Os induction period (day 5–12), and markedly decreased at day 22 (Figure 1B). After day 36, expression of *Sox9* was slightly increased by Os induction but not beyond the expression level at day 12. In contrast, *Sox9* expression was markedly increased by Os-Chon induction at day 36 and further increased until day 43. *Collagen 2a1* (*Col2a1*) expression was significantly increased after day 36 by both Os and Os-Chon induction. After day 36, *Col2a1* expression with Os-Chon induction was much higher than that with Os induction (Figure 1B). Expression of *aggrecan* was significantly, but slightly, increased until day 43 by Os induction (Figure 1B). In contrast, Os-Chon induction dramatically upregulated *aggrecan* after 36 days.

#### 2.1.3. Expression of Pluripotency Marker Genes

Expression of *Nanog* was markedly decreased by Os induction by day 12, to an undetectable level (Figure 1C). Faint expression of *Nanog* was observed at day 22 of Os induction, but gradually disappeared with Os and Os-Chon inductions after day 36. Both Os and Os-Chon induction significantly downregulated *Oct3/4* after 36 days (Figure 1C). Downregulation of *Nanog* and *Oct3/4* after day 36 was more extensive with Os-Chon induction than with Os induction.

### 2.2. Effects of Os Induction on Mineralization and Cartilage Formation in Osteogenically Induced iPSC (OI-iPSC) Constructs

**Figure 2:**
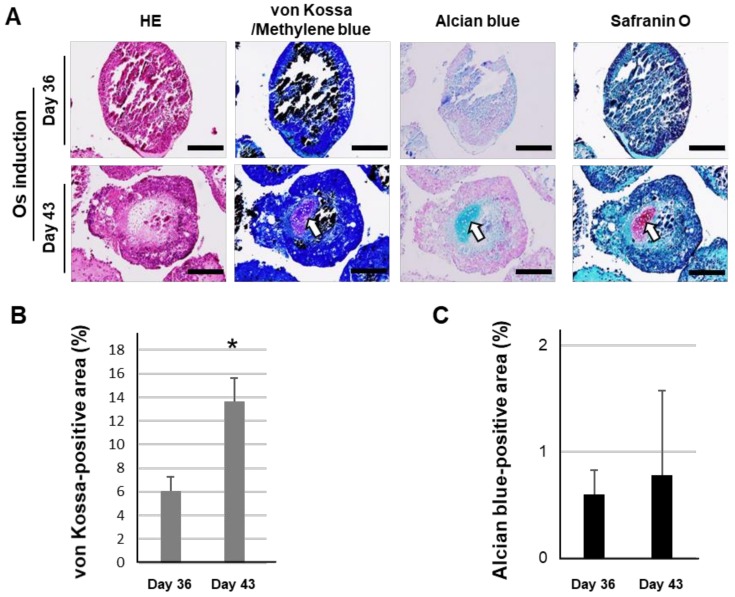
Effects of Os induction on mineralization and cartilage formation in osteogenically induced iPSC (OI-iPSC) constructs. (**A**) Representative images of histochemical staining of OI-iPSC constructs on days 36 and 43 by hematoxylin and eosin (HE), von Kossa (methylene blue-counterstain), alcian blue and safranin O. Arrows indicate cartilage-like tissues. Scale bars; 200 μm. Histomorphometric analysis of (**B**) von Kossa staining-positive (black stained) areas and (**C**) alcian blue-positive areas in the sections. The data represent the mean ± SD (B and C; *n* = 5). Asterisks indicate significant differences between groups (*p* < 0.05, Student’s *t*-test).

Hematoxylin and eosin (HE) staining showed that OI-iPSC constructs at day 36 and 43 had a 2-layer structure, consisting of a large area of unstructured cell mass surrounded by multilayered cells (Figure 2A). von Kossa staining showed robust mineralization (stained in black) in the inner area of OI-iPSC constructs at day 36 and 43. Interestingly, OI-iPSC constructs after day 36 contained some cartilage-like tissue containing large rounded or oval cells, indicated as blue, red and purple-colored areas stained by alcian blue, safranin O and methylene blue, respectively. Histomorphometric analysis of the sections showed that the von Kossa-positive area in OI-iPSC constructs was significantly increased at day 43 by Os induction (Figure 2B). In contrast, the cartilage-like tissue was not significantly different between days 36 and day 43 with Os induction (Figure 2C).

### 2.3. Effects of Os-Chon Induction on Mineralization and Cartilage Formation in Osteo-Chondrogenically Induced iPSC (OCI-iPSC) Constructs

**Figure 3:**
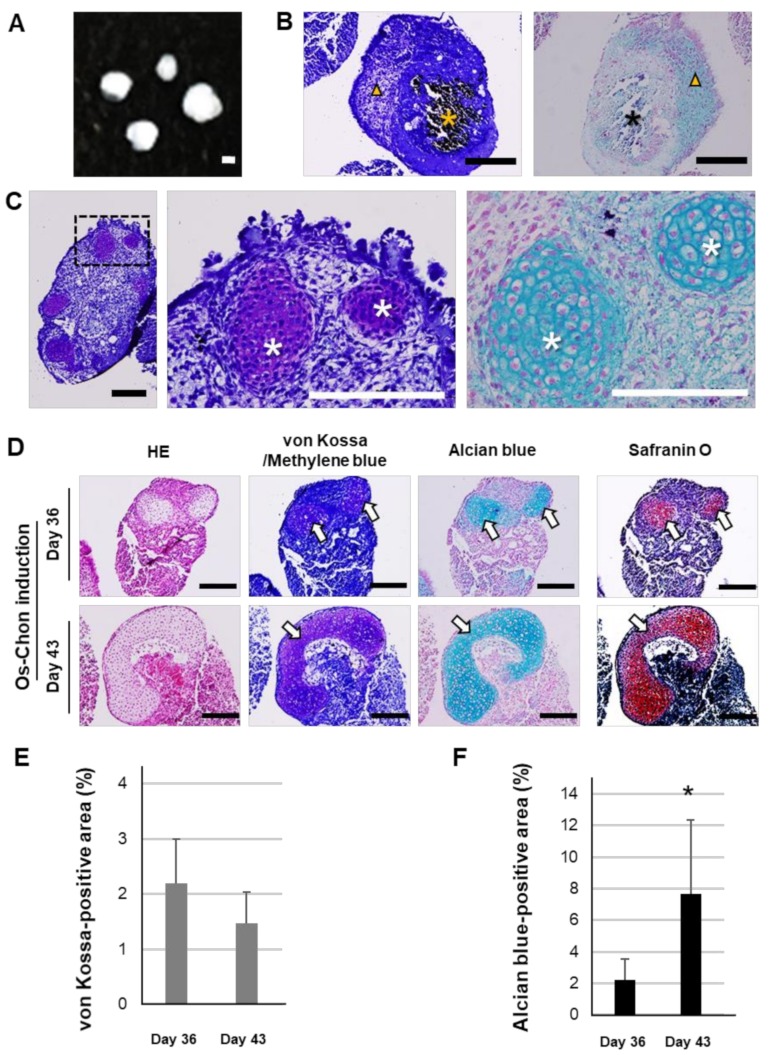
Effects of Os-Chon induction on mineralization and cartilage formation in osteo-chondrogenically induced iPSCs (OCI-iPSCs) constructs. (**A**) OCI-iPSC constructs on day 36. Scale bar; 200 μm. (**B**) Histochemical staining with von Kossa and methylene blue (left panel) and alcian blue (right panel). Asterisks indicate mineralized areas. Arrowheads indicate chondrogenic cell areas. Scale bars; 200 μm. (**C**) Histochemical staining of OCI-iPSC constructs on day 36 with methylene blue (left and middle panels) and alcian blue (right panel). Middle and right panels show magnifications of the dotted square in the left panel. Asterisks indicate cartilage-like tissues. Scale bars; 200 μm. (**D**) Representative images of histochemical staining of OCI-iPSC constructs on days 36 and 43 with HE, von Kossa (methylene blue-counterstain), alcian blue and safranin O. Arrows indicate cartilage-like tissues. Scale bars; 200 μm. Histomorphometric analysis of (**E**) von Kossa staining-positive (black-stained) areas and (**F**) alcian blue-positive areas. The data represent the mean ± SD (E and F; *n* = 5). Asterisks indicate significant difference between groups (*p* < 0.05, Student’s *t*-test).

On visual inspection, OCI-iPSC constructs at day 36 had a white ball morphology (Figure 3A). Histochemical analysis showed mineralization in the inner region and chondrogenic cells in the outer region of OCI-iPSC constructs, which were positively stained with von Kossa and methylene blue/alcian blue, respectively (Figure 3B). Cartilage-like tissues appeared predominantly in the outer region, which contained a group of round cells in lacunae with chondrocyte morphology (Figure 3C). HE staining showed that OCI-iPSC constructs at day 36 and 43 had areas with ECM clearly separated from areas with cartilage-like tissues (Figure 3D). The cartilage-like tissues were positively stained with alcian blue, safranin O and methylene blue. A few von Kossa-positive black areas were observed in OCI-iPSC constructs at days 36 and 43. Histomorphometry showed no significant differences in von Kossa-positive area between days 36 and 43 (Figure 3E). The alcian blue-positive cartilage-like tissue area in OCI-iPSC constructs was significantly increased at day 43 (Figure 3F).

### 2.4. Effects of Os and Os-Chon Induction on the Ratio of Calcification and Cartilage Formation in 3D-iPSC Constructs

**Figure 4:**
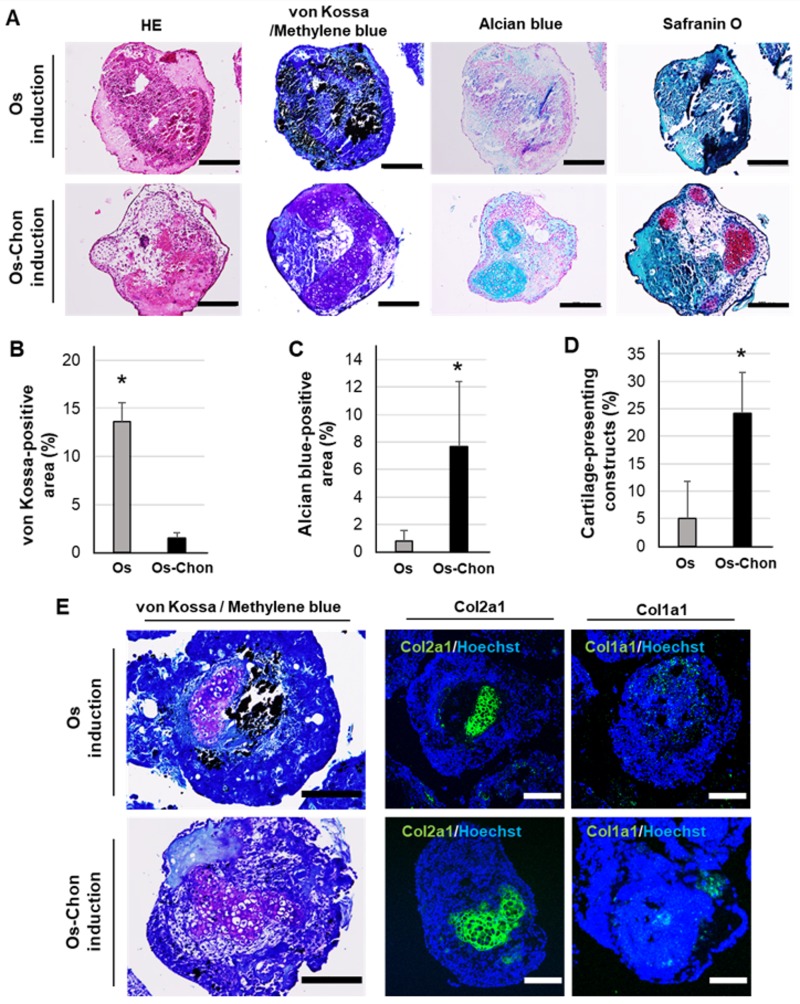
Comparative analysis of bone- and cartilage-like tissue ratio in OI-iPSC and OCI-iPSC constructs at day 43. (**A**) Representative images of histochemical staining by HE, von Kossa (methylene blue-counterstain), alcian blue and safranin O. Scale bars; 200 μm. Histomorphometric analysis of (**B**) von Kossa-positive areas and (**C**) alcian blue-positive areas, and (**D**) proportion of cartilage-containing constructs in OI-iPSC (Os) and OCI-iPSC (Os-Chon) constructs. The data represent the mean ± SD (B–D; *n* = 5). Asterisks indicate significant differences between groups (*p* < 0.05, Student’s *t*-test). (**E**) Representative images of methylene blue-counterstained von Kossa staining and immunohistochemical staining with Col2a1 and Col1a1 in OI-iPSC and OCI-iPSC constructs (nuclear counterstain with Hoechst). Scale bars; 200 µm.

The area ratios of mineralized bone-like tissue and cartilage-like tissue at day 43 in OI-iPSC constructs were compared with those in OCI-iPSC constructs. von Kossa staining showed robust mineralization in OI-iPSC constructs, whereas only a few positive dotted areas were observed in OCI-iPSC constructs (Figure 4A). Histochemical staining with alcian blue, safranin O and methylene blue showed large areas of cartilage-like tissue in OCI-iPSC constructs, whereas positively stained areas were not evident in OI-iPSC constructs. Histomorphometric analysis confirmed a significantly higher ratio of von Kossa-positive area in OI-iPSC constructs than in OCI-iPSC constructs (Figure 4B). The ratio of alcian blue-positive area in OCI-iPSC constructs was much higher than that in OI-iPSC constructs (Figure 4C). The proportion of cartilage-containing constructs at day 43 was approximately 25% in OCI-iPSC constructs, which was significantly higher than that in OI-iPSC constructs (approximately 5%) (Figure 4D).

Methylene blue-counterstained von Kossa staining showed robust mineralization and a small cartilage-like area in OI-iPSC constructs, whereas sparse mineralization and extensive cartilage-like tissue were observed in OCI-iPSC constructs at day 43 (Figure 4E). Immunohistochemistry showed that the methylene blue-positive cartilage-like tissues were positively stained for Col2a1 in both OI-iPSC and OCI-iPSC constructs. Positive staining with Col1a1 was clear in OI-iPSC constructs, whereas weak expression of Col1a1 was observed in OCI-iPSC constructs.

### 2.5. Effects of Os and Os-Chon Induction on Mesodermal Lineage Commitment in 3D-iPSC Constructs

#### 2.5.1. Early Mesodermal Marker Expression

**Figure 5:**
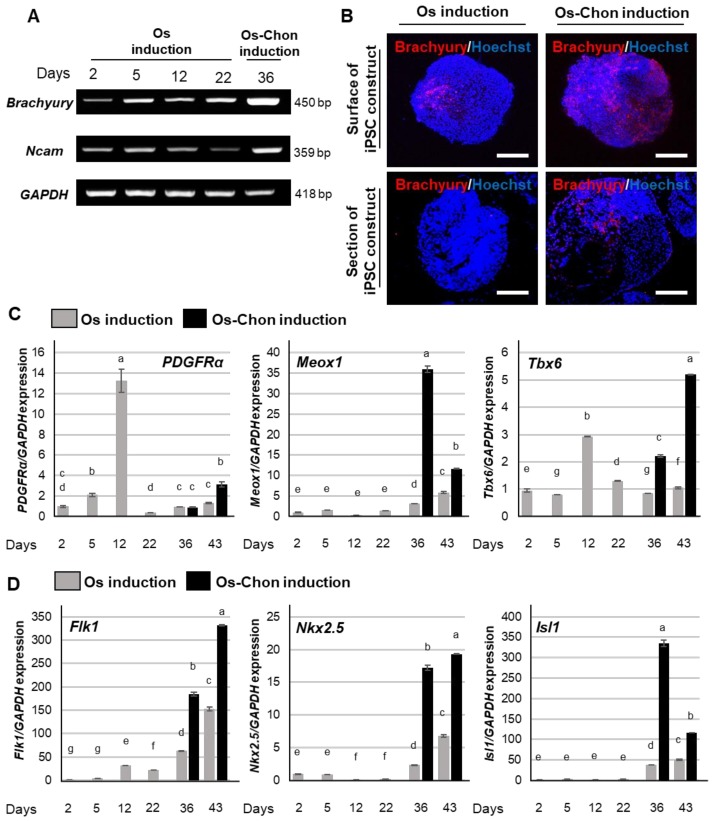
Effects of Os and Os-Chon induction on mesodermal lineage commitment in 3D-iPSC constructs. (**A**) Expression of early mesodermal marker genes, *brachyury* and *Ncam*, was evaluated by semi-quantitative RT-PCR analysis on day 2 (the day when the induction commenced), on day 5, 12, and 22 (Os induction; same induction protocol as Os-Chon induction during this period) and on day 36 (Os-Chon induction). *GAPDH* expression was used as an internal control. (**B**) Representative images of immunohistochemical staining for brachyury on the surface (upper panels) and in cross-sections (lower panels) of OI-iPSC and OCI-iPSC constructs at day 36 (nuclear counterstain with Hoechst). Scale bars; 200 µm. Expression of (**C**) paraxial mesoderm marker genes (*PDGFRα*, *Meox1* and *Tbx6*) and (**D**) lateral plate mesoderm marker genes (*Flk1*, *Nkx2.5 and Isl1*) was evaluated by real-time RT-PCR. The data represent the mean ± SD (*n* = 3). Different letters indicate significant differences between groups (*p* < 0.05, one-way ANOVA with Tukey’s multiple comparison test). *GAPDH* expression was used as an internal control.

Expression of markers for the earliest stage of mesoderm commitment, including *brachyury* [17] and *neural cell adhesion molecule* (*Ncam*) [18], increased in the early stage of Os induction at day 5, and then decreased at day 12 (Figure 5A). Expression of *brachyury* and *Ncam* was increased again at day 36 by Os-Chon induction. Immunohistochemical staining for brachyury showed robust expression on the surface of both OI-iPSC and OCI-iPSC constructs (Figure 5B). The expression in OCI-iPSC constructs was obviously higher than that in OI-iPSC constructs. Many inner cells of OCI-iPSC constructs also expressed brachyury. 

#### 2.5.2. Paraxial Mesoderm (PM) Marker Genes

Expression of *platelet-derived growth factor receptor alpha* (*PDGFRα*) was significantly increased in the early Os induction period (day 5–12), and then decreased at day 22 with sustained weak expression (Figure 5C). Os-Chon induction slightly increased *PDGFRα* expression at day 43. Expression of *mesenchyme homeobox 1* (*Meox1*) was slightly increased after day 36 by Os induction, whereas Os-Chon induction greatly enhanced *Meox1* expression after day 36. Expression of *T-box 6* (*Tbx6*) peaked at day 12 during Os induction. Os-Chon induction resulted in upregulation of *Tbx6* after 36 days.

#### 2.5.3. Lateral Plate Mesoderm (LM) Marker Genes

Expression of *fetal liver kinase 1* (*Flk1*) continuously increased over the Os induction period (Figure 5D). Os-Chon induction greatly upregulated *Flk1* after 36 days. *NK2 Homeobox 5 (Nkx2.5)* was downregulated in the early to middle Os induction period (day 12 and 22), and then upregulated in the late Os and Os-Chon induction period (day 36 and 43). Expression of *LIM domain homeobox gene islet 1* (*Isl1*) was not significantly altered by Os induction until day 22, and was then increased after day 36 by both Os and Os-Chon induction. The increase in the expression of *Nkx2.5* and *Isl1* by Os-Chon induction after day 36 was significantly higher than that by Os induction.

## 3. Discussion

Self-organizing tissues/organs derived from iPSCs provide a unique system to examine mechanisms of organ development and diseases, which could be useful for drug screening [19]. Takebe et al. [8] successfully produced massive and reproducible liver bud organoids from human iPSCs by developing an omni-well-array plate for a micro-space culture environment, which we used in this study. We similarly showed that the omni-well-array allowed iPSCs to form massive and homogeneous 3D spheres, in this case generating osteo-chondrogenic constructs.

The self-organizing potential of iPSCs is expected to facilitate fabrication of tissue-engineered bone/cartilage. Mesenchymal tissues, including bone and cartilage, are generated by mesoderm (PM and LM) and neuroectoderm-derived neural crest [20] (Figure S1). Although it is unclear how RA precisely regulates the lineage commitment of iPSC, RA was previously applied to osteogenic induction protocols for mouse [15,16,21] and human iPSCs [22]. In addition, RA-treated EBs formed from mouse embryonic stem cells (ESCs) produce pre-somatic mesoderm and neural crest cells, both of which provide immature mesenchymal cells that can differentiate into osteoblasts and chondrocytes [23]. Therefore, in this study, we attempted to guide the 3D-iPSC spheres to differentiate into mesenchymal precursor cell constructs by RA treatment.

In the mesodermal development process, PM, which expresses PDGFRα, Tbx6, and Meox1 [24,25], gives rise to derivatives including vertebral bone and vertebral joint cartilage. In contrast, LM, which expresses Flk1, Nkx2.5, and Isl1 [24,25], gives rise to limb bone and limb joint cartilage (Figure S1). Here, we found increased expression of *brachyury* upon RA treatment for 3 days, followed by significant upregulation of several mesoderm marker genes, such as *PDGFRα*, *Tbx6* and *Flk1* in the early stage of Os induction, suggesting mesodermal lineage commitment by our induction protocol. Our work appears to contradict a previous study suggesting that RA treatment of mESC EBs resulted in marked reduction overall mesoderm formation [23]. This discrepancy likely resulted from differences in the 3D sphere formation methods, which considerably affect the characteristics of iPSC spheres for differentiation [26]. Bone and cartilage tissues are also generated by neural crest in the craniofacial region, which is derived from ectoderm-derived neuroectoderm. In our study, iPSC spheres at the early induction stage (day 5) still substantially expressed the pluripotent marker genes *Nanog* and *Oct3/4*, which could partly be explained by the existence of remaining undifferentiated cells. Additionally, Nanog and Oct3/4 play important roles in mouse neural crest stem cell formation [27]. Because RA treatment of ESC EBs produces not only mesodermal cells but also neural crest cells [23], the expression of these genes in iPSC spheres induced by RA treatment might represent differentiation of a portion of the cell population into neural crest cells. Taken together, our results in the early induction stage suggest that RA treatment of iPSC EBs in micro-space culture contributes mesoderm lineage commitment and possible neural crest cell formation.

Mesenchymal condensation, i.e., densely packed aggregation of mesenchymal cells, occurs during the development of almost all tissues, including cartilage and bone [9]. Mesenchymal condensation is initiated by a signaling pathway involving cell adhesion molecules, such as Ncam. In chick limb buds, Ncam is present prior to mesenchymal cell condensation, and its abundance increases during cell aggregation [28]. After the initiation stage, *Sox9* is upregulated during the growth stage of condensation [29]. In our study, increased expression of *Ncam* was observed at the early stage (day 5), along with the gradual upregulation of *Sox9* (day 5–12), which was nearly consistent with the expression pattern of these genes in the mesenchymal condensation process.

In this study, some 3D-iPSC spheres in the omni-well array plate seemed to undergo cell death after day 12, possibly because of nutrient deficiency and hypoxia in the limited micro-space during osteogenic differentiation. We previously reported a method of gentle shaking culture to fabricate in vitro osseous-like tissue from mouse iPSC aggregates [16]. To explore the further self-organization of iPSC constructs, we shifted from the micro-space culture to the shaking culture after day 12. Under the shaking culture condition, Os induction greatly enhanced the expression of the osteogenic marker genes *Osx* and *Ocn* after 36 days. The transcriptional factor Osx plays an important role in osteoblast differentiation [30] and bone mineralization [31]. The calcium binding protein Ocn is one of the most abundant non-collagenous proteins of the bone ECM; thus, it is often used as a mature osteoblast marker [32]. Upregulation of these genes could be indicative of steady osteogenesis of the OI-iPSC constructs. Indeed, OI-iPSC constructs showed a two-layered structure, with a calcified inner region surrounded by an osteoblastic cell layer with abundant ECM including Col1a1. Mechanical force plays an essential role in cell condensation [33] as well as osteogenesis [34]. Our results imply that shaking culture after condensation by micro-space culture could provide appropriate conditions for self-organization of iPSCs into bone-like tissue.

At the late induction stage, Os induction markedly upregulated the LM markers *Flk1*, *Nkx2.5*, and *Isl1* but not PM marker genes, suggesting that Os induction at the late stage guided OI-iPSC constructs to differentiate into limb bud mesenchyme, which provides osteochondral tissue [35] (Figure S1). Interestingly, Os induction under shaking culture enhanced expression of the chondrogenic marker genes *Sox9*, *Col2a1*, and *aggrecan*. These data support previous reports indicating that chondrogenesis is dynamically regulated by mechanical force [10]. The transcription factor Sox9 is essential in mesenchymal condensation and subsequent differentiation during cartilage formation [36]. Col2a1 is a cartilaginous ECM molecule and also expressed by skeletal stem/progenitor cells, including chondrogenic and osteogenic lineage progeny [37]. Expression of aggrecan, which is the major proteoglycan in cartilage tissue [38], and glycosaminoglycan deposition are enhanced by application of dynamic loading in 3D culture of mesenchymal stem cells [39]. Although the appearance of cartilage-like tissue at the late stage of OS induction was rare in the present study, the osteochondrogenic phenotype of OI-iPSC constructs would be stimulated by the combined environment of intrinsic mesenchymal condensation and shaking culture condition.

Osteochondral tissue has a distinctive interfacial zone between bone and cartilage, which plays a crucial role in maintaining cartilage tissue [1]. We found that chondrogenic induction of iPSC spheres after conditioning culture in Os medium greatly enhanced the expression of not only chondrogenic markers (*Sox9*, *Col2a1*, *aggrecan*, and *Ncam*), but also the osteogenic markers *Col1a1* and *Osx*. Although Col1a1 is recognized as a major component of bone ECM and is expressed in osteoblasts, it also plays a role as an important ECM molecule for early commitment of mesenchymal cells toward the chondrogenic lineage [40], which supports the present results. From another point of view, endochondral ossification is regulated by several critical transcription factors, including Sox9 and Osterix [41]. Osterix plays an essential role in the coupling of terminal cartilage differentiation and endochondral ossification in mandibular condylar [42]. In addition, Yang et al. [43] demonstrated that hypertrophic chondrocytes could trans-differentiate to osteoblasts and osteocytes by expressing Col1a1 and Osx during osteochondral bone formation. Although Ncam importantly regulates mesenchymal condensation during chondrogenesis [9], it also highly expresses in preosteoblastic cells during endochondral ossification [28,44]. Therefore, significant upregulation of *Col1a1*, *Osx* and *Ncam* by the chondrogenic induction at the late stage would be partly explained by occurrence of endochondral ossification in a mature chondrogenic cell population in OCI-iPSC constructs.

It seemed that both OI-iPSC and OCI-iPSC constructs at the late induction stage contained cells with maintained stemness because faint expression of *Nanog* and *Oct3/4* was observed during the shaking culture period. Application of shear stress in the presence of LIF synergistically maintains and increases the pluripotency of mouse ESCs [45]. Although the mechanism is not clear, hydrodynamic stress by the shaking culture may partly be involved in the re-expression of *Nanog* and the faint expression of Oct3/4. However, Hu et al., [46] reported that hypertrophic chondrocytes regain stem cell-like properties during endochondral fracture healing through expression of pluripotency-associated transcription factors, including Nanog and Oct3/4, which may also explain our data. Nevertheless, the existence of cells with stem cell-like properties in this study explains the further regulation of mesodermal marker genes in iPSC constructs at the late stage of induction. Indeed, the expression of *brachyury* and both PM and LM marker genes was increased particularly in OCI-iPSC constructs, rather than OI-iPSC constructs, at the late stage (after day 36). Overexpression of brachyury has been reported to accelerate chondrogenesis in mesenchymal stem cells [47]. Os-Chon induction is likely to stimulate the remaining mesodermal progenitors in the iPSC constructs to differentiate into sclerotome and limb-associated chondrogenic cells by upregulating PM and LM marker genes, respectively. The preferential expression of brachyury in the chondrogenic iPSC constructs compared to the osteogenic iPSC constructs at day 36 also supports this possibility.

Taken together, these results suggest that differentiation processes of iPSC constructs induced by Os and Os-Chon in this study are nearly consistent with the development process of bone and cartilage with an osteochondrogenic axis. This suggests that our induction protocol would facilitate self-organization of iPSCs into simplified osteochondral organoids. Bone or cartilage phenotype in the OI-iPSC and OCI-iPSC constructs could be quantitatively shown by histomorphometric analysis. Using these induction methods as a platform, the ratio of osteogenic and chondrogenic regions in the iPSC constructs could be more specifically manipulated by tuning the culture conditions, such as induction periods, medium components and shaking frequency. Although our findings were obtained using mouse iPSCs, this is the first report to demonstrate self-organization of osteochondral tissues directly from iPSC EBs, which would provide a unique system to examine mechanisms of tissue and organ development. In addition, these findings could represent a key step for bioengineering of bone/cartilage hybrid organoids using human iPSCs in the future.

## 4. Materials and Methods

### 4.1. iPSC Culture

Mouse gingival fibroblast-derived iPSCs, which we previously generated using retroviral introduction of Oct3/4, Sox2, and Klf4 (without c-Myc) [48], were used in this study. As described previously [48], iPSCs were expanded on mitomycin C-inactivated SNLP76.7-4 feeder cells using growth medium (ES medium), which consisted of Dulbecco’s modified Eagle’s medium (DMEM with 4.5 g/L glucose and without sodium pyruvate; Nacalai Tesque, Kyoto, Japan), 15% FBS (Thermo Fisher Scientific, Waltham, MA, USA), 2 mM L-glutamine (Wako Pure Chemical, Osaka, Japan), 1 × 10^−4^ M nonessential amino acids (Thermo Fisher Scientific), 1 × 10^−4^ M 2-mercaptoethanol (Thermo Fisher Scientific), and penicillin (50 U)/streptomycin (50 µg/mL) (Wako Pure Chemical).

### 4.2. Fabrication of 3D-iPSC Spheres

iPSCs were maintained on mitomycin C-inactivated SNLP76.7-4 feeder cells in 6-well culture plates (Greiner Bio-One, Frickenhausen, Germany) using ES medium until the cells reached confluence. Then, trypsinization was performed by applying 500 µL of 0.25% trypsin and 1 mM EDTA (Wako Pure Chemical) to each well to first remove SNLP76.7-4 feeder cells. Then, 500 µL of trypsin solution was newly added to iPSCs with gentle pipetting for cell dissociation. Aliquoted single cell suspensions in ES medium were prepared a concentration of 1.95 × 10^6^ cells/mL. The amount of viable iPSCs was measured using an EVE^TM^ automatic cell counter (Nano EnTek, Guro-gu, Seoul, Korea) together with trypan blue staining (Nano EnTek, Guro-gu).

**Figure 6:**
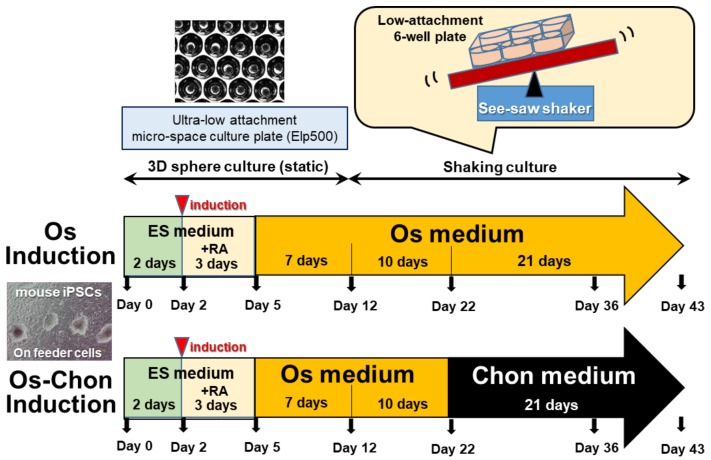
Schematic diagram of the culture protocol for Os and Os-Chon induction of iPSCs (see text for details).

3D-iPSC spheres were fabricated using ultra-low-attachment 24-well micro-space cell culture plates (Figure 6), which contain hundreds of U-bottom-shaped microwell spots per well [8]. The aperture diameter of each microwell dimple is 500 µm and the depth is 700 µm (Elp500; Kuraray, Tokyo, Japan). Then, 1 mL of ES medium was added to each well, and the plates were subsequently subjected to plate-spin-down centrifugation (PlateSpinII, KUBOTA, Tokyo, Japan) to remove the bubbles at the bottom of the micro-space. Next, 1 mL of cell suspension (1.95 × 10^6^ cells/mL) was applied to each well, and cells were maintained for 2 days in ES medium to form 3D-iPSC spheres (EBs).

### 4.3. Osteogenic Induction of 3D-iPSC Constructs

After 2 days of sphere formation, the medium was replaced by ES medium supplemented with 1 µM all trans retinoic acid (RA; Wako Pure Chemical) [21] and was maintained for another 3 days to induce predominantly mesenchymal precursor cells [23]. At day 5 of induction, the culture medium was replaced by osteogenic induction medium (Os medium), consisting of α-MEM (Nacalai Tesque) supplemented with 15% FBS (Thermo Fisher Scientific), 0.1 µM dexamethasone (Sigma-Aldrich, St. Louis, MO, USA), 10 mM β-glycerophosphate (Sigma-Aldrich), 50 µg/mL ascorbate-2-phosphate (Sigma-Aldrich), and 1% antibiotic-antimycotic solution (100 U/mL penicillin, 100 μg/mL streptomycin, and 250 ng/mL amphotericin B; Thermo Fisher Scientific) [15]. The cell constructs were maintained in Elp500 for a total of 12 days, and then iPSC constructs were transferred to a low-attachment 6-well culture plate (Thermo Fisher Scientific) and subjected to shaking force and maintained in Os medium for another 31 days. The transfer ratio was 3 wells of Elp500 to 1 well of non-adherent 6-well plate. The shaking rate used in this study was 0.5 Hertz (Hz) [16]. This induction method and the obtained iPSC constructs were referred to as Os induction and OI-iPSC constructs, respectively, in this study (Figure 6) Medium changes were performed every 2 days, with one-half medium change in Elp500 and full medium change in non-adherent 6-well plates.

### 4.4. Osteochondrogenic Induction of 3D-iPSC Constructs

For Os-Chon induction (Figure 6), Os medium was replaced by chondrogenic induction medium (Chon medium) at day 22, and the culture was maintained for another 21 days under 0.5 Hz of shaking force to obtain OCI-iPSCs. The Chon medium was changed every 2 days. The Chon medium used in this study contained DMEM (with 4.5 g/L glucose and without sodium pyruvate; Nacalai Tesque) supplemented with 10 ng/mL TGFβ-3 (Oncogene Research Products, Cambridge, MA, USA), 100 nM dexamethasone (Sigma-Aldrich), 50 µg/mL ascorbic acid (Sigma-Aldrich), 100 µg/mL sodium pyruvate (Sigma-Aldrich), 40 µg/mL L-proline (Sigma-Aldrich), ITS-plus (Collaborative Biomedical Products, Cambridge, MA; final concentration: 6.25 µg/mL bovine insulin, 6.25 µg/mL transferrin, 6.25 µg/mL selenous acid, 5.33 µg/mL linoleic acid, and 1.25 µg/mL bovine serum albumin) [49].

### 4.5. Reverse Transcription Polymerase Chain Reaction (RT-PCR) Analysis

iPSC constructs were frozen using liquid nitrogen prior to TRIzol extraction (Thermo Fisher Scientific). Then, the RNA was isolated and purified using a spin column (RNeasy Mini Kit; Qiagen, GmBH, Germany) followed by DNase treatment and removal (DNA-free Kit; Thermo Fisher Scientific). Complementary DNA was synthesized from 1 µg of total RNA as previously described [16]. PCR was performed on a StepOnePlus real-time PCR system (Thermo Fisher Scientific) using Thunderbird SYBR qPCR Mix (Toyobo, Osaka, Japan). Expression of target genes was quantitatively analyzed using the ∆∆CT method and normalized against *GAPDH*. The primer sequences used for real-time RT-PCR are shown in Table S1. For semi-quantitative RT-PCR, genes of interest were amplified on a thermal cycler (GeneAtlas G02; Astec Co., Ltd., Fukuoka, Japan) using GoTaq Green Master Mix (Promega Corporation, Madison, WI, USA) according to the manufacturer’s instructions. The primer pairs, cycle number, and annealing temperature used for semi-quantitative RT-PCR are displayed in Table S2. The PCR amplification consisted of an initial denaturation for 5 min at 95 °C, followed by 30 s of denaturation at 95 °C, annealing for 30 s and extension for 30 s at 72 °C. The final extension step was performed at 72 °C for 10 min. Then, PCR products were analyzed using 2% agarose gel electrophoresis containing ethidium bromide. Subsequently, the bands for target genes were detected under ultraviolet (UV) light illumination. *GAPDH* was used as an internal control.

### 4.6. Histochemical Analyses

iPSC constructs were fixed with 10% neutral buffered formalin solution (Wako Pure Chemical) for 1 day prior to paraffin embedding. Sequential sections were prepared with 8 μm thickness using a microtome (Yamato Kohki, Saitama, Japan). Subsequently, the sections were evaluated using histochemical and immunofluorescent staining. The paraffin-embedded sections were deparaffinized with xylene (Wako Pure Chemical) and hydrated through graded alcohol solutions: 100%, 95%, and 70% to distilled water prior to staining.

For HE staining, the section slides were stained with hematoxylin (Sigma-Aldrich) for 5 min, and then washed with running water for 10 min. Next, the slides were stained with eosin (Muto Pure Chemical, Tokyo, Japan) for 2 min. For alcian blue staining, the sections were incubated in 3% acetic acid solution for 5 min prior to staining with alcian blue solution (Wako Pure Chemical) for 30 min. Then, the samples were washed with running water before counterstaining with nuclear fast red solution (Sigma-Aldrich) for 5 min. For safranin O staining, the specimens were stained for 10 min with Weigert’s iron hematoxylin working solution prepared from a 1:1 mixing ratio of Weigert’s iron hematoxylin solution I (Muto Pure Chemical) and solution II (Muto Pure Chemical). After the sections were washed in running water for 10 min, the sections were stained with 0.05% fast green solution for 5 min. Then, the sections were rinsed with 1% acetic acid solution for less than 10–15 s prior to staining in 0.1% safranin O solution for 5 min. For staining, 0.05% fast green solution and 0.1% safranin O solution were prepared from fast green powder (Wako Pure Chemical) and safranin O powder (Waldeck GmbH & Co KG, Münster, Germany), respectively. For methylene blue-counterstained von Kossa staining, the specimens were incubated with 5% silver nitrate solution (AgNO_3_; Wako Pure Chemical) under UV light for 10 min, and then rinsed in two changes of distilled water. Next, the slides were incubated in 5% sodium thiosulfate (Sigma-Aldrich) for 5 min to eliminate un-reacted silver, and then washed with running water for 5 min. Subsequently, the sections were stained with 1% methylene blue (Wako Pure Chemical) in 10 mM borate buffer for 30 min. After staining, all slides were dehydrated with graded ethanol solutions (70%, 95%, and 100%), and cleared in xylene. Finally, the specimen slides were covered with coverslips using mounting medium (Malinol; Muto Pure Chemicals) for further observation.

### 4.7. Histomorphometric Quantification

Areas positive for alcian blue staining and von Kossa staining were quantified from randomly selected images of iPSC constructs at days 36 and 43 (*n* = 5) in 200× magnified sections from each specimen using ImageJ software (National Institutes of Health, Bethesda, MD, USA). The alcian blue-positive area was calculated as the ratio of the stained area (threshold: 95) to the whole construct area. The area positive for von Kossa staining was evaluated as the ratio of the stained area (threshold: 100) to the whole construct area.

### 4.8. Immunohistochemical Staining

Immunofluorescent staining of iPSC constructs was performed after 1 day of fixation using 10% neutral buffered formalin solution (Wako Pure Chemical). The cell constructs were washed with PBS prior to incubation in blocking buffer consisting of 2% BSA (Wako Pure Chemical), 0.1% Tween20 (Sigma-Aldrich), and 0.01% Triton-X (Wako Pure Chemical) for 60 min to block non-specific binding sites and for permeabilization. Then, the cell constructs were incubated in anti-brachyury polyclonal antibody (AF2085: 10 μg/mL, R&D Systems, Minneapolis, MN, USA) at 4 °C overnight in a humid chamber. Then, the constructs were washed with PBS for 5 min twice prior to incubation in Alexa Fluor 555-conjugated donkey anti-goat IgG (1/500, Molecular Probes, Invitrogen, Eugene, OR, USA) for 60 min at room temperature.

For immunofluorescent staining of deparaffinized sections, rehydration was performed through a graded alcohol series to distilled water. Then, antigen retrieval was performed via incubation of specimens in 0.1% pepsin (Nacalai Tesque) in 0.5 M acetic acid (Sigma-Aldrich) at 37 °C for 1 h in a humid chamber followed by conventional immunofluorescent staining. The primary antibodies used for staining were anti-type I collagen monoclonal antibody (NB600-450: 1/50, Novus Biologicals, Littleton, CO, USA), anti-type II collagen monoclonal antibody (sc-52658: 1/50, Santa Cruz Biotechnology, CA, USA) and anti-brachyury polyclonal antibody (AF2085: 10μg/mL, R&D Systems). The secondary antibodies used in this study were Alexa Fluor 488-conjugated goat anti-mouse IgG (1/500, Molecular Probes, Thermo Fisher Scientific) or Alexa Fluor 555-conjugated donkey anti-goat IgG (1/500, Molecular Probes, Invitrogen). Nuclear staining was performed using Hoechst 33258 (1/500, Thermo Fisher Scientific). Subsequently, fluorescent staining of target proteins was assessed using a fluorescence microscope (LSM780, Zeiss).

### 4.9. Statistical Analyses

One-way analysis of variance (ANOVA) with Tukey post hoc test was used for a comparison of more than two groups. Student’s *t*-test was performed for the comparison of two groups. Statistical significance was defined as *p* < 0.05.

## 5. Conclusions

This study established a platform for induction of mouse iPSCs to osteochondral constructs using micro-space culture followed by shaking culture. In this platform, the bone and cartilage ratio in the iPSC constructs could be manipulated simply by selecting either osteogenic or chondrogenic medium during shaking culture. Fabrication of osteochondral tissues from mouse iPSCs by following the developmental stages, as demonstrated in this study, could be advantageous to develop human iPSC-derived bone/cartilage organoids. Therefore, our findings are promising for the development of iPSC-based bone/cartilage organoids for regenerative therapy, disease modeling and drug screening.

## Supplementary Materials

Supplementary materials can be found at https://www.mdpi.com/1422-0067/21/2/581/s1.
