# Supplementary material for: In Vitro Fabrication of Hybrid Bone/Cartilage Complex Using Mouse Induced Pluripotent Stem Cells
Source: Int J Mol Sci. 2020 Jan 16;21(2):581. doi: 10.3390/ijms21020581 (PMC7014254; doi:10.3390/ijms21020581)
Supplement: Supplementary file 1 [file ijms-21-00581-s001.pdf]

International Journal of *Molecular Sciences*

Table S1. Primers used for SYBR Green real-time RT-PCR.

Description (Gene Name)	Primers (Fw, Forward; Rv, Reverse)	bp	Accession Number
Osterix (*Sp7*)	Fw: 5'-CTCGTCTGACTGCCTGCCTAG-3'<br>Rv: 5'-GCGTGGATGCCTGCCTTGTA-3'	84	NM_130458.3
Collagen 1a1 (*Col1a1*)	Fw: 5'-TGTCCCAACCCCCAAAGAC-3'<br>Rv: 5'-CCCTCGACTCCTACATCTTCTGA-3'	92	NM_007742.3
Osteocalcin (*Bglap*)	Fw: 5'-CCGGGAGCAGTGTGAGCTTA-3'<br>Rv: 5'-AGGCGGTCTTCAAGCCATACT-3'	68	NM_007541.3
Sox9 (*Sox9*)	Fw: 5'-CCTTCAACCTTCCTCACTACAGC-3'<br>Rv: 5'-GGTGGAGTAGAGCCCTGAGC-3'	131	NM_011448.4
Aggrecan (*aggerecan*)	Fw: 5'-CGCCACTTTCATGACCGAGA-3'<br>Rv: 5'-TCATTCAGACCGATCCACTGGTAG-3'	146	NM_007424.2
Collagen 2a1 (*Col2a1*)	Fw: 5'-CCTCCGTCTACTGTCCACTGA-3'<br>Rv: 5'-ATTGGAGCCCTGGATGAGCA-3'	121	NM_001113515.2
Flk1 (*Kdr*)	Fw: 5'-GGCGGTGGTGACAGTATCTT-3'<br>Rv: 5'-CTCGGTGATGTACACGATGC-3'	198	NM_010612.2
Nkx2.5 (*Nkx2.5*)	Fw: 5'-CAGTGGAGCTGGACAAAGCC-3'<br>Rv: 5'-TAGCGACGGTTCTGGAACCA-3'	217	NM_008700.2
Isl1 (*Isl1*)	Fw: 5'-GCGCTCATGAAGGAGCAACTA-3'<br>Rv: 5'-TGATGCTGCGTTTCTTGTCC-3'	103	NM_021459.4
PDGFRα (*Pdgfra*)	Fw: 5'-TACATCATCCCCCTGCCAGA-3'<br>Rv: 5'-AAGGTTATCCCGAGGAGGCT-3'	270	NM_001083316.2
Meox1 (*Meox1*)	Fw: 5'-GGAAGGAGAGGACAGCCTTC-3'<br>Rv: 5'-CCCTTCACACGTTTCCACTT-3'	178	NM_010791.3
Tbx6 (*Tbx6*)	Fw: 5'-CCTGACTCTCCTGCCACTG-3'<br>Rv: 5'-CCTCTTCACACGGGCATCC-3'	339	NM_011538.2
GAPDH (*Gapdh*)	Fw: 5'- TGCACCACCAACTGCTTAG-3'<br>Rv: 5′- GGATGCAGGGATGATGTTC-3'	177	NM_001289726.1

Table S2. Primers used for semi-quantitative RT-PCR.

Description (Gene Name)	Primers (Fw, Forward; Rv, Reverse)	bp	Ann Temp	Cycles	Accession Number
Ncam (*Ncam1*)	Fw: 5′-CTCCCTGCCTCCAACCATCATC-3′<br>Rv: 5′-TCTCGTCATCTTCCTCCTCGTTCTC-3′	359	65 °C	32	NM_001081445.1
Brachyury (*T*)	Fw: 5′-CCAGCTCTAAGGAACCACCG-3′<br>Rv: 5′-TGTCCACGAGGCTATGAGGA-3′	450	61 °C	36	NM_009309.2
Nanog (*Nanog*)	Fw: 5′-AGGGTCTGCTACTGAGATGCTCTG-3′<br>Rv: 5′-CAACCACTGGTTTTTCTGCCACCG-3′	363	70 °C	30	NM_028016.3
Oct3/4 (*Oct3/4*)	Fw: 5′-TCTTTCCACCAGGCCCCCGGCTC-3′<br>Rv: 5′-TGCGGGCGGACATGGGGAGATCC-3′	224	67 °C	35	NM_013633.3

GAPDH *(Gapdh)*	Fw: 5'-CACCATGGAGAAGGCCGGGG-3' Rv: 5′-GACGGACACATTGGGGGTAG-3'	418	60 °C	26	NM_001289726.1

Ann Temp: annealing temperature.

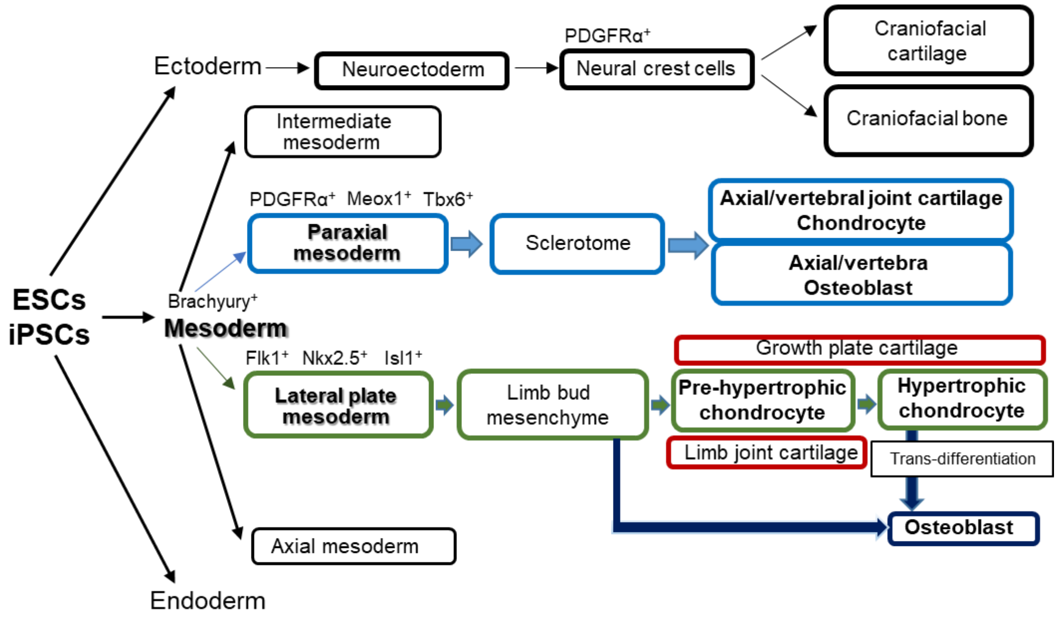

Figure S1. Schematic diagram of establishment of osteoblast and chondrocyte lineages from pluripotent stem cells. The diagram is produced by reference articles [20,35,43].
